# MoVi: A large multi-purpose human motion and video dataset

**DOI:** 10.1371/journal.pone.0253157

**Published:** 2021-06-17

**Authors:** Saeed Ghorbani, Kimia Mahdaviani, Anne Thaler, Konrad Kording, Douglas James Cook, Gunnar Blohm, Nikolaus F. Troje

**Affiliations:** 1 Department of Electrical Engineering and Computer Science, York University, Toronto, ON, Canada; 2 Centre for Vision Research, York University, Toronto, ON, Canada; 3 Department of Psychology, Queen’s University, Kingston, ON, Canada; 4 Department of Biology, York University, Toronto, ON, Canada; 5 Departments of Neuroscience and Bioengineering, University of Pennsylvania, Philadelphia, Pennsylvania, United States of America; 6 Centre for Neuroscience Studies, Queen’s University, Kingston, ON, Canada; 7 Department of Surgery, Queen’s University, Kingston, ON, Canada; University of Innsbruck, AUSTRIA

## Abstract

Large high-quality datasets of human body shape and kinematics lay the foundation for modelling and simulation approaches in computer vision, computer graphics, and biomechanics. Creating datasets that combine naturalistic recordings with high-accuracy data about ground truth body shape and pose is challenging because different motion recording systems are either optimized for one or the other. We address this issue in our dataset by using different hardware systems to record partially overlapping information and synchronized data that lend themselves to transfer learning. This multimodal dataset contains 9 hours of optical motion capture data, 17 hours of video data from 4 different points of view recorded by stationary and hand-held cameras, and 6.6 hours of inertial measurement units data recorded from 60 female and 30 male actors performing a collection of 21 everyday actions and sports movements. The processed motion capture data is also available as realistic 3D human meshes. We anticipate use of this dataset for research on human pose estimation, action recognition, motion modelling, gait analysis, and body shape reconstruction.

## 1 Introduction

Capturing, modelling, and simulating human body shape and kinematics has been an area of intense study in the fields of biomechanics, computer vision, and computer graphics, with applications including human-machine interactions [1], assistive healthcare [2], clinical diagnostics [3], and realistic computer animation pipelines [4–6]. In order to obtain body pose and kinematics at a resolution that is fine enough to make inferences about identity, action, and particularly stylistic features, we need large, high-quality datasets that can be used in both generative and discriminative contexts. An unsolved challenge is to create datasets that combine video recordings of humans in motion in unconstrained scenarios with information on ground truth about the dynamic pose and shape of the recorded individuals.

Research in computer vision has focused on understanding humans and their behaviour from images or videos. Obtaining reliable, high-accuracy data about the “true” pose and shape and its changes over time, however, requires sensors that might interfere with the ecological validity of the image or video. For instance, optical motion capture has the potential to provide 3D pose and body shape [7], but conflicts with wearing normal clothing, leaves visible markers in the video, and can only be used in a laboratory environment. Other sensors, such as inertial measurement units (IMU), can be hidden under clothing and are feasible to capture humans in natural settings, but do not provide absolute location information and suffer from drift. One approach to eliminating this drift in IMU data is to detect the 2D joints of the body in a simultaneously recorded video [8]. Thus, limitations of one hardware system can partially be overcome by combining it with recordings of another.

No available single hardware system is able to capture people in a natural setting and simultaneously provide high precision ground truth data of body shape and pose. All publicly available datasets suffer from this limitation to some degree [9–13]. Some are also limited in that they either contain data of only a small number of different actors, use single hardware systems for motion recording, or provide unsynchronized data across different hardware systems. We address these limitations in our dataset by providing subsets of data with partially overlapping information that lend themselves to transfer learning. Our dataset contains five different subsets of synchronized and calibrated video, optical motion capture, and IMU data. Each subset features the same 90 female and male actors performing the same set of 20 predefined everyday actions and sports movements, plus one self-chosen movement.

An important advantage of our dataset is that the full-body motion capture recordings are also available as realistic 3D human meshes represented by a rigged body model as part of the AMASS database [7]. Because we recorded the same actors with varying combinations of sensors, these animated meshes can also be used as ground truth body shape for the recording subsets with sparse markers and natural clothing. In addition to the MoSh++ formulation used in AMASS, we calculated the skeletal pose using the biomechanics formulation provided by the Visual3D software [14]. The synchronized and calibrated motion capture system and stationary video cameras allow computing and augmenting accurate frame-by-frame overlay of 3D skeletal pose and body surface in camera and motion capture coordinates. For our natural clothing captures, we recorded the motions using IMU sensors and video cameras, with and without additional sparse motion capture markerset. The sparse optical markerset could be combined with the IMU data to accurately extract end-effector locations and infer body pose.

This multi-modal dataset is designed for a variety of challenges including gait analysis, human pose estimation and tracking, action recognition, motion modelling, and body shape reconstruction from monocular video data and different points of view. To our knowledge, this is one of the largest datasets in terms of the recorded number of actors and performed actions, and the first dataset with synchronized pose, pose-dependent and pose-independent body shape, and video recordings. The fact that we recorded the same actions from the same actors with varying combination of sensors makes our dataset unique.

## 2 Methods

### 2.1 Subjects

90 people (60 women, 30 men) with no reported neurological or musculoskeletal conditions that affected their ability to perform common sports movements were recruited from the local Kingston community. Participant characteristics are provided in Table 1. The experimental procedure was approved by the General Research Ethics Board of Queen’s University, Kingston, and was performed in accordance with the Declaration of Helsinki. All participants provided written informed consent that their data (including their video footage) can be used by other researchers. The two individuals depicted in this manuscript gave written informed consent (as outlined in PLOS consent form) to include their photographs in this publication.

**Table 1:** Participant characteristics of the 60 women and 30 men.

Women						Men					
ID	Age	Height [cm]	Weight [kg]	BMI [kg/m^2^]	Handedness	ID	Age	Height [cm]	Weight [kg]	BMI [kg/m^2^]	Handedness
2	33	152	54	23.37	right	1	25	184	92	27.17	right
6	26	155	59	24.56	right	3	26	167	59	21.16	right
7	22	175	73	23.84	right	4	26	178	80	25.25	right
8	22	160	52	20.31	right	5	23	180	73	22.53	right
9	23	157	48	19.47	right	11	27	178	90	28.41	right
10	24	175	63	20.57	right	13	26	178	77	24.30	right
12	26	162	68	25.91	right	15	21	181	72	21.98	right
14	21	157	61	24.75	right	18	25	170	65	22.49	right
16	26	163	68	25.59	right	19	18	167	60	21.51	left
17	26	167	65	23.31	right	20	29	173	60	20.05	right
21	21	160	55	21.48	right	22	28	170	66	22.84	right
24	20	160	55	21.48	right	23	25	173	73	24.39	right
25	21	166	55	19.96	right	26	24	178	63	19.88	right
30	19	178	68	21.46	right	27	23	163	64	24.09	right
32	20	168	57	20.20	right	28	25	183	80	23.89	right
34	21	155	41	17.07	left	29	24	177	61	19.47	right
38	32	157	53	21.50	right	31	28	175	64	20.90	right
39	21	175	77	25.14	right	33	21	175	60	19.59	right
40	21	175	56	18.29	right	35	29	176	72	23.24	right
44	20	162	75	28.58	right	36	29	174	74	24.44	left
45	18	165	48	17.63	right	37	21	169	63	22.06	right
48	18	144	68	32.79	right	41	28	178	100	31.56	right
49	23	155	45	18.73	right	42	21	165	63	23.14	right
50	18	155	59	24.56	right	43	21	175	80	26.12	right
51	18	167	63	22.59	right	46	21	188	84	23.77	right
52	20	162	54	20.58	right	47	18	175	80	26.12	left
53	23	179	60	18.73	right	60	21	178	73	23.04	right
54	18	165	70	25.71	right	71	18	173	59	19.71	right
55	20	161	62	23.92	right	75	19	162	86	32.77	right
56	19	176	72	23.24	right	87	18	185	76	22.21	right
57	17	170	61	21.11	right						
58	18	158	52	20.83	right						
59	18	170	68	23.53	right						
61	18	167	74	26.53	right						
62	17	177	69	22.02	right						
63	18	160	58	22.66	right						
64	18	165	49	18.00	right						
65	19	174	58	19.16	right						
66	18	162	50	19.05	right						
67	18	174	59	19.49	right						
68	20	174	57	18.83	right						
69	19	161	65	25.08	right						
70	17	178	68	21.46	right						
72	20	158	60	24.03	right						
73	18	162	57	21.72	right						
74	19	171	61	20.86	right						
76	19	164	61	22.68	right						
77	19	170	63	21.80	right						
78	18	150	46	20.44	right						
79	19	168	77	27.28	right						
80	19	155	70	29.14	right						
81	18	165	59	21.67	left						
82	17	168	59	20.90	right						
83	18	178	61	19.25	right						
84	20	165	63	23.14	right						
85	19	174	64	21.14	right						
86	18	168	59	20.90	right						
88	19	168	57	20.20	right						
89	21	165	54	19.83	right						
90	32	165	58	21.30	right						

### 2.2 Acquisition setup

**Fig 1:**
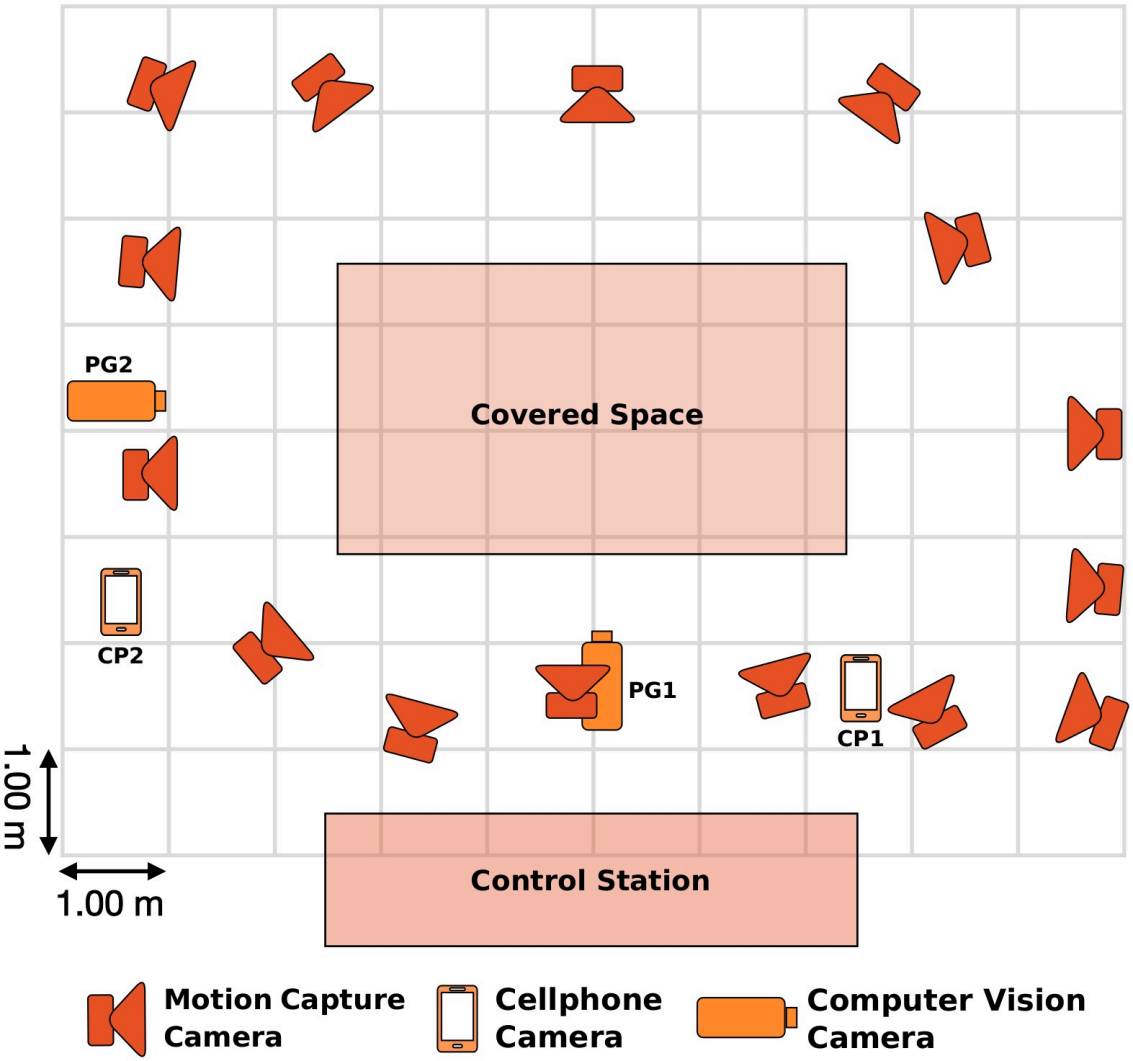
Top view sketch of the capture room set-up. The positions of the video cameras and motion capture cameras were arranged to cover a space of approximately 3 by 5 meters.

An optical motion capture system, stationary and hand-held video cameras, and inertial measurement unit (IMU) sensors were used to record the dataset. Fig 1 shows the top-view floor plan of the capture room with the motion capture and video cameras arranged to cover a space of approximately 3 by 5 meters to allow subjects to perform their movements without spatial restrictions. In the following sections, the details of the hardware and software systems along with their calibration and synchronization process details are provided.

#### 2.2.1 Hardware and software systems

*2.2.1.1 Optical motion capture system*. 15 Qualisys Oqus 300 and 310 cameras (Qualisys AB, Sweden, https://www.qualisys.com/) were used. The cameras were set to normal mode (full field of view) with a resolution of 1.3 MP and captured the 3D location of passive reflective markers of 0.7 cm diameter with a frame rate of 120 frames per second (fps). The Qualisys Track Manager (QTM) software was used for the acquisition of the optical motion capture data and for setting the synchronization triggering signal that was sent to the Grasshopper video cameras that were connected to the motion capture system.

*2.2.1.2 Video cameras*. Video data were collected using two hand-held smartphone cameras and two stationary computer vision cameras. For the hand-held cameras, the rear camera of the iPhone 7 (Apple Inc., USA, https://www.apple.com/) was used. The camera has a resolution of 1920 × 1080 pixels and contains the Sony IExmor RS, CMOS sensor. The video data was recorded with a frame rate of 30 fps. As computer vision cameras, we used RGB Grasshopper2 cameras (FLIR Systems Inc., USA, https://www.flir.com/) with a resolution of 800 × 600 pixels, 72 dpi, 24-bit depth and Sony ICX285 CCD sensors. The recording with these cameras was also done with a frame rate of 30 fps. The FlyCapture software provided by FLIR Inc. was used for setting up the cameras’ acquisition features and for processing the synchronization triggering signal coming from the motion capture system. We also integrated the MATLAB Image Acquisition Toolbox as it supports the Grasshopper computer vision cameras and provides blocks and functionalities such as hardware triggering, configuring acquisition parameters and recorded data format, and previewing the recorded data.

**Fig 2:**
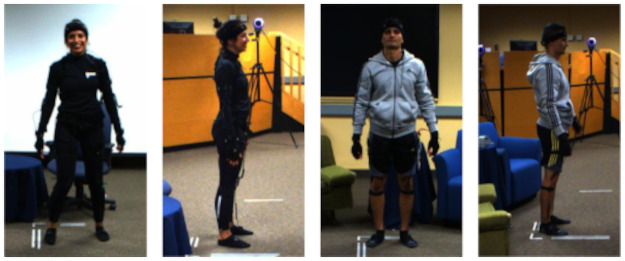
Example pictures of one female and one male actor wearing the IMU suits used for the capture rounds S1, S2, I1, and I2.

**Fig 3:**
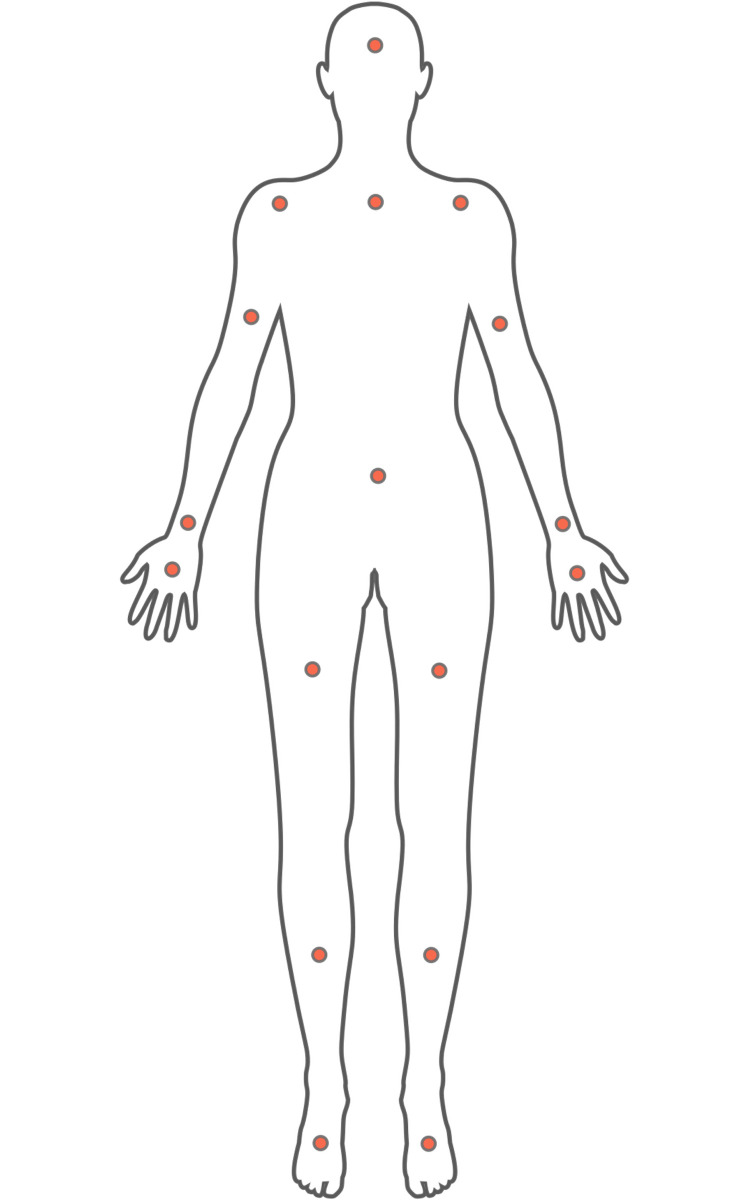
Placement of IMU sensors on the body.

*2.2.1.3 Inertial measurement unit sensors*. The Noitom Neuron Edition V2 (Noitom LTD, China, https://www.noitom.com/) was used which comes as a bodysuit attached with 17 IMU sensors (Figs 2 and 3). Each sensor is composed of a 3-axis gyroscope, 3-axis accelerometer, and 3-axis magnetometer working at 120 Hz. In addition to the acceleration data, the IMU suit provides computed 3D displacements, velocity, quaternions, and rotational velocity for each joint (all represented in an initial global coordinate system). The IMU sensors’ dynamic range, accelerometer range, and gyroscope range are 360 *deg*, ±16 *g*, and ±2000 *deg*/*s*, respectively. The static error of the sensors is less than 1 *deg* for all roll, pitch, and yaw angles. The AXIS NEURON software provided by Noitom LTD was used for setting the acquisition features, calibration of the sensors, data capturing, validation of the recorded data, and for exporting the files to different formats.

### 2.3 Data collection

Participants went through five data capturing sequences. The sequences differed in the hardware systems used to capture the motions, in participants’ clothing (minimal, or normal), and whether or not there was a rest pose between successive motions. An overview of the different capture rounds is provided in Table 2. In each sequence, participants performed the same predefined set of 20 movements in a randomized order and additionally one self-chosen movement, based on verbal instructions by the experimenter. The movements included everyday actions and sports movements: (1) walking, (2) jogging, (3) running in place, (4) side gallop, (5) crawling, (6) vertical jumping, (7) jumping jacks, (8) kicking, (9) stretching, (10) crossing arms, (11) sitting down on a chair, (11) crossing legs while sitting, (13) pointing, (14) clapping hands, (15) scratching one’s head, (16) throwing and catching, (17) waving, (18) taking a picture, (19) talking on the phone, (20) checking one’s watch, (21) performing a self-chosen movement. To allow for more variation in performed movements in each action class, we did not set any constraints on how exactly each action had to be performed.

**Table 2:** Overview of the five different capture rounds.

Data Capture Sequence	F	S1	S2	I1	I2
Motion capture markerset	67	12	12	–	–
Video capture	yes	yes	yes	yes	yes
IMU	no	yes	yes	yes	yes
A-pose between motions	yes	yes	no	yes	no
Actor clothing	minimal	normal clothing	normal clothing	normal clothing	normal clothing
Length (min per person)	∼2.7	∼2.7	∼1.7	∼2.7	∼1.7

F = full motion capture markerset, S = sparse motion capture markerset + IMU, I = IMU; 1 = with rest A-pose, 2 = without rest A-pose.

#### 2.3.1 Data capture sequence “F”

This sequence was captured using the 67 MoSh motion capture marker layout [15]. Subjects wore tight-fitting minimal clothing in order to minimize marker movement relative to the body. The markers were attached to the actors’ skin and clothes using double-sided tape. In addition to the motion capturing, video material was recorded using two stationary Grasshopper cameras and the rear cameras of two hand-held iPhones 7. For details on the synchronization of the motion capture system and the stationary cameras, see Synchronization Section. Participants performed the actions separated by a rest A-pose. The motivation for this capture round was to obtain accurate full skeletal (pose) information and frame-by-frame body shape parameters without any artefacts imposed by clothing. Therefore, this round is suitable for 2D or 3D pose estimation and tracking, and 3D shape reconstruction.

#### 2.3.2 Data capture sequences “S1” and “S2”

For these two sequences, subjects wore the IMU bodysuit and a reduced optical motion markerset layout of 12 motion capture markers that were attached to their body (4 markers placed on the head, 2 on each ankle and 2 on each wrist). In addition, the actions were recorded using synchronized computer vision cameras (see Synchronization section), and iPhone 7 rear cameras. In “S1” there was a rest A-pose between the actions, whereas in “S2” there was a natural transition between the performed actions. The reason for choosing a small motion capture markerset was that it provides sparse, but accurate data for some of the main end-effectors including the head, wrists, and ankles, and at the same time allows participants to wear natural clothing.

#### 2.3.3 Data capture sequences “I1” and “I2”

These two sequences were captured with participants wearing the IMU suit under their normal clothing. Additionally, video material was recorded using the hand-held iPhone 7 and stationary Grasshopper video cameras. Motions in “I1” are separated by a rest A-pose, whereas there is a natural transition between the actions in “I2”.

### 2.4 Preprocessing

#### 2.4.1 Motion capture data

A cubic polynomial gap filling was automatically done in the QTM software for gaps of less than or equal to 5 frames. The trajectories were then labelled manually using the integrated trajectory identification tool. The resulting labelled trajectories were then exported to a C3D format.

#### 2.4.2 Video data

Each data capture sequence was recorded in one piece, without stopping the recording between the different actions. Therefore, the recorded sequences by the computer vision cameras were manually trimmed into individual single actions and the time-stamps (frame numbers) of start and end of each action were exported. Based on the time-stamps, the corresponding synchronized motion capture and IMU data were also trimmed into the same individual single actions.

#### 2.4.3 IMU data

The original IMU data stored in calculation file format (.calc) were re-organized and converted into MATLAB .mat files to get the data in a more readable structure.

### 2.5 Calibration

#### 2.5.1 Motion capture cameras

The calibration of the motion capture cameras was done before each recording session following the measurement protocol in the Qualisys Track Manager software [16]. The software allows computing the orientation and position of each camera in order to track and perform calculations on the 2D data for conversion into 3D data. The average residual error of the calibration was kept below 0.8 mm and the calibration was repeated if this threshold was not met.

#### 2.5.2 Video cameras

To compute the intrinsic parameters of the Grasshopper computer vision cameras and lens distortion parameters, the MATLAB Single Camera Calibrator [17–19] was used, where focal length ($F\in R^{2}$), optical center ($C\in R^{2}$), skew coefficient (S∈R), and radial distortion ($D\in R^{2}$) are estimated for each camera. The average re-projection error was kept to less than 0.2 pixel, and the calibration was repeated for higher error values. No calibration was performed for the iPhone cameras.

#### 2.5.3 IMU device

*2.5.3.1 Model posture calibration*: Before starting each session, a four-step calibration process was required to calibrate the actor’s posture. The four-step calibration process is performed by the actor posing in a steady pose, A pose, T pose, and S pose.

*2.5.3.2 Neuron calibration*: IMU sensors might accumulate some calculation errors over time. This usually causes posture computation problems such as drifting. Therefore, each individual IMU sensor should be calibrated after some time of usage. However, to make sure that recordings are accurate enough, we calibrated the sensors before collecting data from each subject following the Noitom Axis Neuron user manual [20].

#### 2.5.4 Motion capture and video cross-calibration

To cross-calibrate the motion capture system with the two Grasshopper computer vision cameras, the location of world points was aligned onto the camera coordinates. For that, the extrinsic parameters which represent the rotation *R* ∈ *SO*(3) and translation $T\in R^{3}$ from the motion capture system’s coordinate system (world coordinates) to the camera coordinates were estimated using the semi-automated method proposed by Sigal et al. [9]. The trajectory of a single moving marker was recorded by the synchronized motion capture and video cameras for > 2000 frames. Given the recorded 3D positions of the marker in motion capture coordinates as world points and the 2D positions of the marker in the camera frame as image points, the problem of finding the best 2D projection can be formulated as a Perspective-*n*-Point (PnP) problem where the Perspective-Three-Point (P3P) algorithm [21] is used to minimize the re-projection error as follows:

$$\begin{array}{c} \underset{R,T}{\text{min}}\sum_{n=1}^{N}{\left\|P_{2D}\left[n\right]-f(P_{3D};R,T,K)\left[n\right]\right\|}^{2}, \end{array}$$ (1)

where *n* is the frame number, *N* > 2000 is the total number of recorded frames, *f* is the projection function and *K* is the set of camera intrinsic and lens distortion parameters. The 2D position of the single marker was located using a Hough circle transform [22] and double-checked manually frame-by-frame.

**Fig 4:**
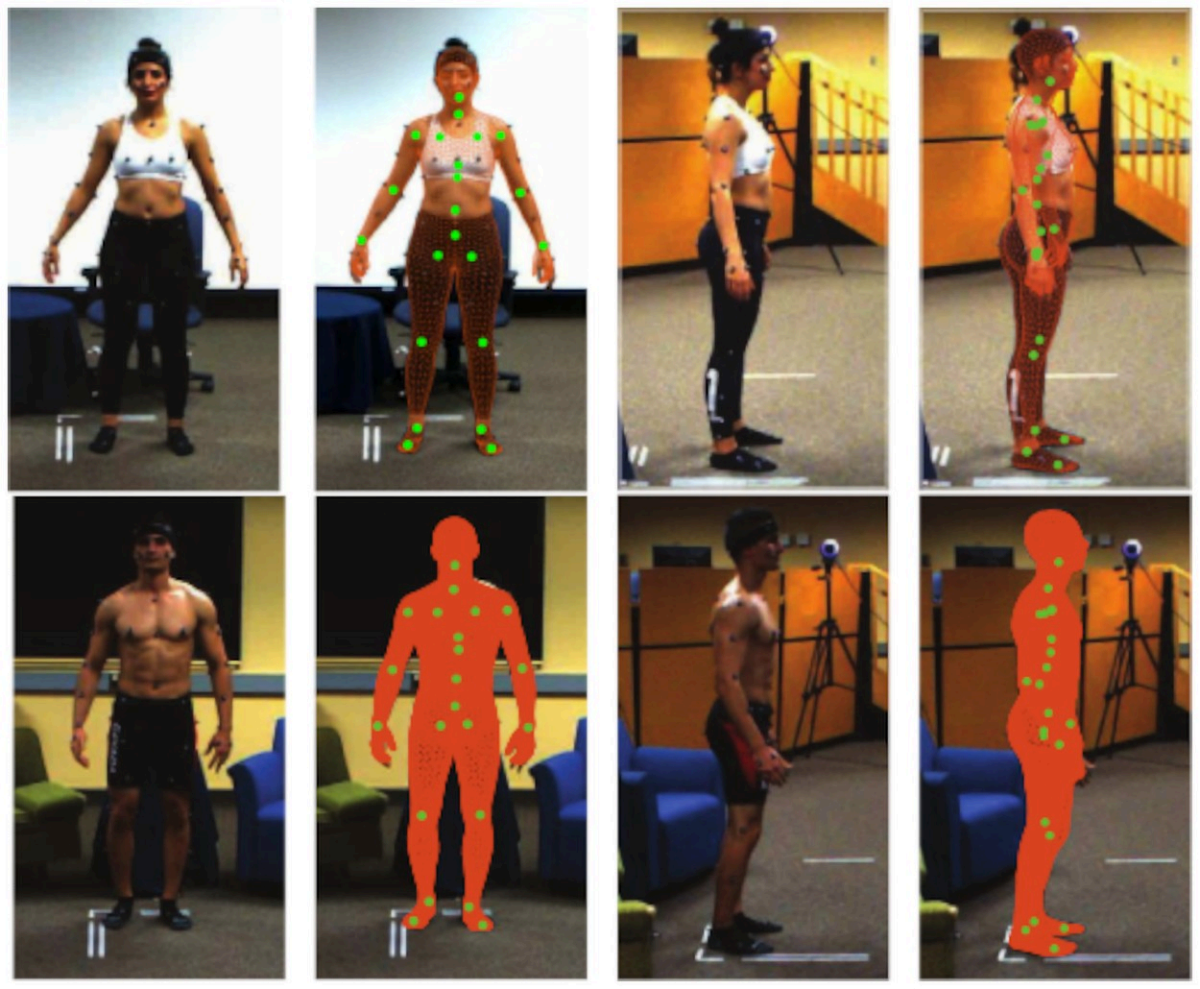
Front and side view of aligned video frame, joint locations, and estimated body mesh (computed by MoSh++) for one female and male participant.

To validate the computed extrinsic parameters, the parameters were evaluated on a separate single marker capture session. The average re-projection RMS error on this test run was around 0.8 cm. Synchronization and calibration were additionally validated by careful visual inspection of the accuracy of overlaid joint and mesh positions on the video data for a random selection of multiple rounds. The examples shown in Fig 4 are representative for the quality of the whole database.

### 2.6 Synchronization

#### 2.6.1 Motion capture and video data

For the data capture sequences “F”, “S1”, and “S2”, the motion capture system and the cameras had to be time-synchronized. In our setup, the video cameras were triggered by the synchronization signal from the QTM software of the motion capture system through ethernet. Due to the frame rate limits in the video cameras, the synchronization frequency was divided by 4 which reduced the video capture frame rate to 30 fps. The iPhone cameras were not synchronized with the motion capture cameras.

#### 2.6.2 Motion capture and IMU data

To use both IMU and motion capture data in the sequences “S1” and “S2” in a data fusion scenario, these modalities needed to be synchronized in frame. To this end, the cross-correlation between the z-axis location of ankles was used which was pre-computed in these two modalities. The two coordinate systems were not aligned, however, the differences between the orientation of the two *z* axes are negligible: the *z* axis of the IMU coordinate system is oriented towards gravity, while *z* axis in motion capture coordinate system is perpendicular to the floor. Because the motion capture system was synchronized with the video cameras, we additionally obtained synchronized IMU and video data.

Suppose $p_{z}^{j}\left[n\right]$ and ${\tilde{p}}_{z}^{j}\left[n\right]$ are the *z* component of tracked position of joint *j* at time-step *n* recovered by the motion capture and IMU systems, respectively (we are using the 3D positions provided by the IMU software instead of double-integrating over accelerations). The synchronization parameters, temporal scale *α* and temporal shift *τ*, are found by maximizing:

$$\begin{array}{c} \underset{\alpha ,\tau }{\text{max}}\sum_{n=-\infty }^{\infty }p_{z}^{j}\left[n\right]{\tilde{p}}_{z}^{j}\left[\alpha n+\tau \right], \end{array}$$ (2)

where the integral is the cross-correlation between $p_{z}^{j}\left[n\right]$ and scaled version of ${\tilde{p}}_{z}^{j}\left[n\right]$. The optimal parameters, by which the highest peak in cross-correlation is achieved, were found using an exhaustive search for 0.9 ≤ *α* ≤ 1.1 (search step-size = 0.001) and −200 ≤ *τ* ≤ 200. The second term in the summation in Eq 2 was evaluated using spline interpolation. We found *α* = 1 for all samples meaning that there was no scaling. To ensure that the optimized parameters were robust, we normalized the resulted cross-correlation (Eq 2) to the maximum of 1 and only accepted those where the distance between first and second peak was higher than 0.3. Only in 3 out of all “S1” and “S2” rounds the parameters got rejected and the synchronization was repeated. Finally, we did a visual inspection of all accepted samples.

### 2.7 Skeleton and body shape extraction

The motion capture data collected in *“F”* was processed using two different pipelines to compute the skeleton: Visual3D [14] (C-Motion Inc., USA, https://c-motion.com/) and MoSh++ [7, 15] (https://amass.is.tue.mpg.de/). The data collected in *“S”* was processed using Visual3D and the same formulas for computing head, wrists, and ankles joint positions. Example images of one female and male participant in rest A-pose with overlaid joint locations and mesh are shown in Fig 4.

#### 2.7.1 Visual3D software

Visual3D is a biomechanics analysis software for 3D motion capture data [14]. In our Visual3D pipeline, the pelvic segment was created using CODA [23] and the hip joint positions were estimated using Bell and Brand’s hip joint center regression [24, 25]. The upper body parts were estimated using the Golem/Plug-in Gait Upper Extremity model [26]. The resulting skeleton at each frame is represented by 20 joints in two different formats: 1) in local joint transformations, that is the orientation and translation of each joint relative to the coordinate system of its parent joint in the kinematic tree, and 2) as global 3D joint locations.

#### 2.7.2 MoSh++

MoSh++ is an approach which estimates the body shape, pose, and soft tissue deformation directly from motion capture data [7]. Body shape and pose are represented using the rigged body model SMPL [4] where the pose is defined by joint angles and shape is specified by shape blend shapes. MoSh++ achieves lower errors compared to the original MoSh framework [15], which used the SCAPE body model [27]. It uses a generative inference approach whereby the SMPL body shape and pose parameters are optimized to minimize reconstruction errors. The skeletal joint locations are computed using a linear regression function of mesh vertices. The estimated SMPL body is extended by adding dynamic blend shapes using the dynamic shape space of DMPL to simulate soft tissue deformations. Each frame in the “MoSh-ed” representation includes 16 SMPL shape coefficients, 8 DMPL dynamic soft-tissue coefficients, and 66 SMPL pose coefficients as joint angles (21 joints + 1 root). MoSh-ed data of our motion capture recordings was computed in collaboration with the authors of AMASS [7].

The main difference between MoSh++ and Visual3D is that the models are optimized for different applications. MoSh++ is a better choice for character animation, and pose estimation and tracking, whereas Visual3D is preferred for gait analyses and biomechanics. MoSh++, on the one hand, can provide an estimate of joint transformations for all joints even if marker occlusion occurs. However, the estimated joint locations can be noisy when occlusions occur and the error may propagate to other joints. This is because MoSh++ uses distributed information by regressing from the inferred body mesh to the skeleton joints. For character animations, however, precise joint locations are often not important. For gait analysis and biomechanics applications, on the other hand, an accurate estimation of joint locations is crucial. Visual3D achieves this by doing the computations locally where each joint location is computed only from the surrounding markers. The only drawback of Visual3D representation compared to MoSh++ is that the joints cannot be computed at all if one of contributing markers is occluded. In the database, we indicated the time-stamps of the frames where such occlusions occurred.

## 3 Data records

Table 3 shows the file structures of the raw and processed data which are provided in the MoVi Dataverse repository [28], with naming conventions and detailed descriptions.

**Table 3:** Naming conventions and structure of all files available in the database. 〈ID〉 ∈ {1,2,…,90} indicates the subject number,〈round〉 ∈ {F,S1,S2,I1,I2} the data collection round, and 〈camera〉 ∈ {PG1,PG2,CP1,CP2} the camera type where PG stands for the computer vision cameras and CP for the cellphone cameras.

Data Type	File Name	Description
Video Data	〈round〉_〈camera〉_Subject_〈ID〉.〈format〉	avi video data from the computer vision cameras (PG1, PG2) for rounds F, S1, and S2, and mp4 video data from the cellphone cameras (CP1, CP2) for all rounds (F, S1, S2, I1, and I2) and all subjects (1-90). Note that we provide code to trim the video sequences to single motion clips for round F.
Camera Parameters	cameraParams_〈camera〉.〈format〉	Contains the camera intrinsic calibration data for camera PG1 and PG2 in .mat, .npz, and .pkl formats. These parameters are fixed for the whole dataset.
	Extrinsics_〈camera〉.〈format〉	Contains the camera extrinsics parameters for camera PG1 and PG2 (rotation matrix and translation vector) in .mat, .npz, and .pkl formats.
Motion Capture Data	F_amass_Subject_〈ID〉.mat	Contains the full markerset motion capture data (round F) processed by MoSh++ in the AMASS project and augmented with 3D joint positions and metadata for each subject (1-90). All files are compressed and stored as F_AMASS.tar. The original npz files and the rendered animation files are available at https://amass.is.tue.mpg.de/. Note that we provide code to trim the motion capture sequences to single motion clips.
	F_v3d_Subject_〈ID〉.mat	Contains the full markerset motion capture data (round F) processed by Visual3D and augmented with metadata for each subject (1-90). All files are compressed and stored as F_Subjects_〈ID〉_〈ID〉.tar as containers of 45 subjects (e.g., ID 1-45, ID 46-90).
	S_v3d_Subject_〈ID〉.mat	Contains the motion capture data from rounds S1 and S2 processed by Visual3D and augmented with metadata. All files are compressed and stored as S_V3D.tar.
IMU Data	imu_Subject_〈ID〉.mat	Contains the processed IMU calculation files augmented with metadata. Each file contains the data collected in all rounds (S1, S2, I1, I2). The files are compressed as IMUmatlab_Subject_〈ID〉_〈ID〉.tar containers of 15 subjects (e.g., ID 1-15, ID 16-30 etc).
	imu_Subject_〈ID〉.bvh	Contains IMU in .bvh format. Each file contains the data collected in all rounds (S1, S2, I1, I2). The files are compressed as IMUbvh_Subject_〈ID〉_〈ID〉.tar containers of 15 subjects (e.g., ID 1-15, ID 16-30 etc).

### 3.1 Raw data

Raw video data from the computer vision cameras is provided as .avi video files to avoid any artefacts added by compression methods. Raw motion capture data stored as .qtm files that are only readable by the QTM software and .c3d and raw IMU data stored in .xml and .calc file formats are not included in the MoVi database. However, these files can be provided by the corresponding author upon request.

### 3.2 Processed data

The processed full markerset motion capture data (capture round “F”) is provided in two different versions based on the post-processing pipeline (MoSh++/AMASS and Visual3D). We provide joint angles and 3D joint locations computed by both pipelines along with the associated kinematic tree, information about the occlusions and optical marker data. Both versions are provided as .mat format for each subject. The .mat file also contains body pose-independent shape parameters provided by the MoSh++ pipeline as SMPL blend shape coefficients [4]. Given pose-independent shape parameters and joint angles, corrective pose-dependent shape parameters and the resulting surface mesh represented as frame-by-frame 3D vertices can be computed. Due to the reduced markerset, the motion capture data collected in rounds “S1” and “S2” were only processed using the Visual3D pipeline for extracting the head, wrists, and ankles’ joint positions provided as .mat files. Synchronized IMU data were computed by processing the .calc files and converting them to .mat format which provides raw acceleration data, displacement, velocity, quaternions, and angular velocity. The .bvh files generated by the IMU software are also provided in the repository.

## 4 Applications

The MoVi dataset is currently the only synchronized and cross-calibrated video, motion capture, and IMU dataset that provides accurate 3D body shape and pose. Importantly, by using different combinations of hardware systems to record the same actors and motions, the dataset provides overlapping information that can facilitate training models for body shape reconstruction, and pose estimation and tracking from video data.

For body shape reconstruction tasks, our dataset provides 3D body shape based on the SMPL model which does not only provide pose-independent shape parameters, but also pose-dependent shape parameters, and therefore allows for more accurate shape representation.

For body pose estimation tasks, our dataset contains two formats of body pose representations based on motion capture data, Visual3D [14] and SMPL/MoSh++ [4, 7]. Visual3D is a biomechanical model that provides accurate estimation of joint locations if no marker occlusions occur, and is therefore suitable for motion modelling and gait analysis. MoSh++ provides an estimate of all joint locations (although noisy when occlusions occur), and is therefore more suitable for pose estimation and tracking tasks. In addition to capture round *F* with full optical marker set, we used a sparse set of optical markers in rounds *S1* and *S2* to reduce the visual artefacts in the video material. The sparse marker set still provides ground truth 3D position of the main joints while still featuring natural clothing. MoVi also provides challenging action types that are useful for training robust pose estimation models, such as cross-legged sitting and crawling, but that are not commonly seen in other datasets with ground truth 3D pose.

The large number of 90 individual actors who performed the same set of actions, provides high diversity across performers in terms of action type, action execution, style, and modalities (video, motion capture, and IMU) which are important factors for research on action recognition (see e.g., [29] who used our dataset for action recognition). This is also important for frameworks for designing character animation that focus on modelling the natural stochasticity and diversity of the movements (e.g, [5]).

## 5 Usage notes

To support easy accessibility and usage of our dataset in different research fields, processed and raw data are provided in the data repository. Not all raw data are part of the MoVi database. However, all raw data can be made available upon request by the corresponding author. Preprocessing code can also be made available to users who are interested in working on raw data or reproducing processed data along with other datasets.

The motion capture and IMU data were processed and organized in .mat file format, in a way that they can be easily used for any of the challenges mentioned above. In the following Github repository, we provide scripts for easy importing of these .mat files into both MATLAB and Python environments: https://github.com/saeed1262/MoVi-Toolbox. In addition to the import scripts, all of the needed scripts for preparation, processing, and visualization are also provided in the Github repository. Detailed instructions on how to access the dataset and the license agreement for using the dataset are provided on the dataset website (https://www.biomotionlab.ca/movi). The original .npz files of the processed motion capture data using the MoSh++ method are provided as part of the AMASS dataset (https://amass.is.tue.mpg.de). AMASS provides a unified environment to integrate and compare our dataset to other existing optical motion capture datasets.

## 6 Code availability

The custom MATLAB and Python scripts for processing the data are provided on the following Github repository: https://github.com/saeed1262/MoVi-Toolbox. The repository contains all the necessary tools for file reading, conversion, processing, and visualization. An additional tutorial is provided on how to use the dataset.

## 7 Summary

The MoVi dataset includes five data subsets that were recorded using synchronized video, optical motion capture and IMU hardware systems to provide partially overlapping information across the different subsets. It features the same 60 female and 30 male actors who repeat the same set of 21 everyday motions and sports movements in each data subset. In total, MoVi contains 9 hours of optical motion capture data, 17 hours of video data recorded from 4 different points of view with both hand-held and stationary cameras, and 6.6 hours of IMU data. To our knowledge, our dataset is the largest dataset in terms of the number of subjects and performed motions, and the first dataset with synchronized pose, pose-dependent and pose-independent body shape, and video recordings.
